# Follow-On Indications for Orphan Drugs Related to the Inflation Reduction Act

**DOI:** 10.1001/jamanetworkopen.2023.29006

**Published:** 2023-08-15

**Authors:** James D. Chambers, Katherine A. Clifford, Daniel E. Enright, Peter J. Neumann

**Affiliations:** 1 Center for the Evaluation of Value and Risk in Health, Tufts Medical Center, Boston, Massachusetts

## Abstract

This cohort study assesses rates at which orphan drugs approved by the US Food and Drug Administration were also approved for supplemental (follow-on) indications.

## Introduction

The Inflation Reduction Act (IRA) requires Medicare price negotiation for selected drugs beginning in 2026.^1^ The law is expected to lower drug prices, but observers have debated the extent to which it will influence future innovation.^2,3^ The IRA exempts orphan drugs^4^ (ie, drugs for rare diseases) from negotiations, although this exemption applies only to orphan drugs with a single approved indication. As a consequence, drug companies will have reduced incentives to pursue additional uses of their orphan products. To inform debates around how the IRA may affect development of treatments for rare diseases, we examined how many orphan drugs approved by the US Food and Drug Administration (FDA) have had supplemental (follow-on) approved indications.

## Methods

We identified novel orphan drug approvals (2003 through 2022) from FDA’s New Molecular Entity (NME) Drug and New Biologic Approvals database.^5^ We reviewed each drug’s FDA label current in April 2023 to determine whether FDA had approved the drug for a follow-on indication, and if so, for how many indications. We categorized each follow-on indication as orphan vs nonorphan, and calculated the time from a drug’s initial approval to the follow-on indication approval. This study was exempt from institutional review by the Tufts Medical Center.

To better understand the types of conditions addressed by follow-on indications, we identified whether the FDA included the approval in 1 or more expedited review programs (which the FDA reserves for therapies targeting serious illness and unmet clinical needs): accelerated approval, priority review, fast track, and/or breakthrough therapy.^6^

## Results

FDA approved 282 novel orphan drugs from 2003 to 2022. FDA labels were unavailable for 2 drugs, leaving 280 drugs for analysis. The FDA approved 63 orphan drugs (23%) for at least 1 follow-on indication, including 29 (10%) for multiple follow-on indications (Table 1). Overall, the FDA approved 152 separate follow-on indications; 92 (61%) of these follow-on indications were also for orphan drug conditions. The mean (SD) time from novel orphan drug approval to follow-on indication was 53 (43) months (median [IQR], 46 [57] months) (Table 2). The FDA included 58 (38%) follow-on indications in one expedited review program, 46 (30%) in 2 programs, and 17 (11%) in 3 programs; none were included in all 4 programs.

**Table 1.** Novel Orphan Drug Approvals and Follow-On Indications

Characteristics	Follow-on indications, No. (%)				
	All years (2003-2022)	2003-2007	2008-2012	2013-2017	2018-2022
Total novel orphan drugs approvals, No.	280	32	47	74	127
Follow-on indications					
None	217 (78)	24 (75)	32 (68)	49 (66)	112 (88)
1	34 (12)	4 (13)	8 (17)	10 (14)	12 (9)
2	14 (5)	2 (6)	2 (4)	7 (9)	3 (2)
3	4 (1)	0	2 (4)	2 (3)	0
≥4	11 (4)	2 (6)	3 (6)	6 (8)	0

**Table 2.** Follow-On Indications for Novel Orphan Drug Approvals

Characteristic	Expedited program approvals, No. (%)		
	Total follow-on indications (N = 152)	Orphan follow-on indications (n = 92)	Nonorphan follow-on indications (n = 60)
Time to follow-on, mean (SD), mo	53.1 (42.5)	56.8 (45.1)	47.3 (37.7)
Time to follow-on, median (IQR), mo	46.4 (18.0-75.3)	48.0 (25.4.9-85.2)	38.8 (2.7-72.0)
Total expedited programs per approved drug			
None	31 (20)	20 (22)	11 (18)
1	58 (38)	34 (37)	24 (40)
2	46 (30)	29 (32)	17 (28)
3	17 (11)	9 (10)	8 (13)
4	0	0	0
Expedited review program			
Priority	89 (59)	57 (62)	32 (53)
Fast-track	17 (11)	9 (10)	8 (13)
Accelerated	42 (27)	23 (25)	19 (32)
Breakthrough^a^	53 (38)	30 (35)	23 (43)

Abbreviation: FDA, US Food and Drug Administration.

^a^ Out of a total 140 FDA-approved follow-on indications after the breakthrough therapy program was established in 2012. Orphan follow-on indications were out of 86 and nonorphan indications out of 54.

## Discussion

Efforts to address prescription drug costs must balance the benefits of lower drug prices with downsides in terms of reduced future innovation. We provide new data to help understand the potential consequences of incentives inherent in the IRA for drug companies to curtail efforts to pursue future follow-on indications for orphan drugs. FDA approved roughly one quarter of orphan drugs from 2003 to 2022 for at least 1 follow-on indication, and the agency considered the majority of these indications in expedited review programs.

How much the IRA will affect future innovation is unknown and a source of controversy. Estimates of how many fewer drugs will be developed are wide-ranging.^2,3^ The law may lead pharmaceutical manufacturers to develop more single-indication orphan drugs (which are not subject to negotiations) rather than follow-on indications. Our analysis suggests that the potential foregone follow-on indication approvals for serious illness and unmet needs could be nontrivial. Such potential losses should be considered against the gains to consumers and society that come with lower drug prices.

Study limitations include that we did not account for the clinical outcomes of follow-on indications, we did not account for the fact that drugs approved for a single use may also be used for off-label indications, and we did not address other incentives created by the IRA.

## Notes

Supplement.
Data Sharing Statement
