# Supplementary material for: Follow-On Indications for Orphan Drugs Related to the Inflation Reduction Act
Source: JAMA Netw Open. 2023 Aug 15;6(8):e2329006. doi: 10.1001/jamanetworkopen.2023.29006 (PMC10427936; doi:10.1001/jamanetworkopen.2023.29006)
Supplement: Supplement. — Data Sharing Statement [file jamanetwopen-e2329006-s001.pdf]

Data Sharing Statement

Chambers. Follow-On Indications for Orphan Drugs Related to the Inflation Reduction Act. *JAMA Netw Open*. Published August 15, 2023. doi:10.1001/jamanetworkopen.2023.29006

Data
Data available: Yes
Data types: Other (please specify)
Additional Information: The data we relied on is available on the FDA website.
How to access data: https://www.fda.gov/
When available: With publication

Supporting Documents
Document types: None

Additional Information
Who can access the data: Please see above.
Types of analyses: Please see above.
Mechanisms of data availability: We will link to the relied upon sources in the manuscript
